# Supplementary material for: How do people with dementia use the ambulance service? A retrospective study in England: the HOMEWARD project
Source: BMJ Open. 2018 Aug 1;8(7):e022549. doi: 10.1136/bmjopen-2018-022549 (PMC6074617; doi:10.1136/bmjopen-2018-022549)
Supplement: Supplementary file 1 [file bmjopen-2018-022549supp001.pdf]

Appendix A: HOMEWARD Phase 1- Coding for SWASFT/ EEAST Data

CODES

Call Colour codes:

1- red
2- green
3- amber- not available in EEAST

See Treat or Convey codes

1- See & Treat
2- See & Convey (include transport only in EEAST)

Qualification codes- identify most senior clinician recorded at scene

1. Technician
2. Paramedic/SWAMP paramedic
3. Lead Paramedic
4. Specialist Paramedic/Critical Care Paramedic
5. Other- contains the following
    a. BLS trained (first responder)
    b. Ambulance Care Assistant
    c. Emergency Care Assistant
    d. Student Paramedic
    e. Operational Officer
    f. Doctor

Gender codes

1- Male
2- Female

Presenting condition codes

**SWASFT**	**EEAST**
1- General	Other
2- Cardiac	AF, Heart Failure, Hypertension, MI, other cardiac
3- Respiratory	Asthma, COPD, other respiratory
4- Endocrine	Diabetes I or II, also other for thyroid
5- Neurological	Stroke/ CVA, TIA, Other neurological
6- Gastro-Intestinal	Other
7- Musculoskeletal	Musculoskeletal
8- Obs/gynae	Other
9- Cancer	Other
10- Mental health	Mental Health
11- Trauma (excluding self-harm)	Trauma
12- Other medical	Other
13- Social	Social
14- Other (any condition not otherwise specified)	Other

15- Deliberate self-harm	Suicide/ self-harm
16- Environmental	Other
(17- Sepsis) see other medical	Other e.g. UTI, Chest Infection
18- Poisoning (accidental)	Other
19- Burns	Other
	Cognitive impairment/ confusion- delirium
	Dementia
	Nil apparent
	Palliative

Provisional diagnosis codes

SWASFT	EEAST
1- General	Other
2- Cardiac	AF, Heart Failure, Hypertension, MI, other cardiac
3- Respiratory	Asthma, COPD, other respiratory
4- Endocrine	Diabetes I or II, also other for thyroid
5- Neurological	Stroke/ CVA, TIA, Other neurological
6- Gastro-Intestinal	Other
7- Musculoskeletal	Musculoskeletal
8- Obs/gynae	Other
9- Cancer	Other
10- Mental health	Mental Health
11- Trauma (excluding self-harm)	Trauma
12- Other medical	Other
13- Social	Social
14- Other (any condition not otherwise specified)	Other
15- Deliberate self-harm	Suicide/ self-harm
16- Environmental	Other
(17- Sepsis) see other medical	Other e.g. UTI, Chest Infection
18- Poisoning (accidental)	Other
19- Burns	Other
	Cognitive impairment/ confusion- delirium
	Dementia
	Nil apparent
	Palliative

Search terms for target and control groups:

Target group:

Dementia, Alzheimer's, Fronto-temporal dementia, vascular dementia, senility, Pick's disease, Lewy Body disease/ dementia

1- Dementia present - Presence of the diagnosis of one of the above
2- Dementia suspected - paramedic suggests dementia may be present eg ? dementia, seeing memory nurse, taking dementia medication
3- Dementia inferred - Paramedic infers ongoing problem through use of other language such mild cognitive impairment, cognitive impairment, memory loss. Sometimes this might also be called confusion but is not acute. This might be confirmed as 'normal or usual for the patient'.

Control group:

Confusion- acute, delirium,

4- Presence of one of the above

Drugs search terms:

Drugs: Acumor (XL), **Aricept**, Axurer, Consium XL, **Donepezil,** Ebixa, Elmino, Exelon, **Galantamine,** Galsya (XL), Gazylan XL, Gatalin (XL), Kerstipon, Lotprosin XL, Luventa XL, Maruxa, **Memantine,** Nemdatine, Nimvastid, Reminyl (XL), **Rivastigmine**, Valios.

PATCHES: Alzest, Eluden, Prometax, Rivatev, Voleze

1- Presence of these drugs in case record

Social History code:

1- lives alone, no care package
2- lives alone, with care package
3- lives with spouse/ partner, no care package
4- lives with spouse/ partner, with care package
5- lives with extended family, no care package
6- lives with extended family, with care package
7- nursing home
8- residential home
9- other

Location codes:

1- Home
2- Nursing home
3- Residential Home
4- Public place
5- Other
6- Not documented

Capacity to consent

1- Patient has capacity
2- Patient does not have capacity

Rockwood Frailty code:

1- Very Fit
2- Well
3- 3 Managing Well
4- Vulnerable
5- Mildly Frail
6- Moderately Frail
7- Severely Frail
8- Very Severely Frail
9- Terminally Ill

Falls

Search conducted in presenting condition and HPC for fall related calls. This is to ensure those coded later as trauma are also coded for falls. Falls are coded as present if they occurred within last 6 days and have contributed to the call.

Search terms fall, fell, slip, trip
