# Supplementary material for: How do people with dementia use the ambulance service? A retrospective study in England: the HOMEWARD project
Source: BMJ Open. 2018 Aug 1;8(7):e022549. doi: 10.1136/bmjopen-2018-022549 (PMC6074617; doi:10.1136/bmjopen-2018-022549)
Supplement: Author's manuscript [file bmjopen-2018-022549.draft_revisions.pdf]

BMJ Open is committed to open peer review. As part of this commitment we make the peer review history of every article we publish publicly available.

When an article is published we post the peer reviewers' comments and the authors' responses online. We also post the versions of the paper that were used during peer review. These are the versions that the peer review comments apply to.

The versions of the paper that follow are the versions that were submitted during the peer review process. They are not the versions of record or the final published versions. They should not be cited or distributed as the published version of this manuscript.

BMJ Open is an open access journal and the full, final, typeset and author-corrected version of record of the manuscript is available on our site with no access controls, subscription charges or pay-per-view fees (http://bmjopen.bmj.com).

If you have any questions on BMJ Open's open peer review process please email info.bmjopen@bmj.com

BMJ Open

The use of emergency ambulance services by people with dementia: The HOMEWARD Project

Journal:	*BMJ Open*
Manuscript ID	bmjopen-2018-022549
Article Type:	Research
Date Submitted by the Author:	28-Feb-2018

Complete List of Authors:	Voss, Sarah; University of the West of England, Health and Life Sciences<br>Brandling, Janet; University of the West of England, Health and Life Sciences<br>Taylor, Hazel; Research Design Service South West,<br>Black, Sarah; South Western Ambulance Service NHS Foundation Trust,<br>Buswell, Marina; University of Hertfordshire, CRIPACC< School of Health and Social Work<br>Cheston, Richard; University of the West of England, Health and Social Sciences<br>Cullum, Sarah; University of Auckland<br>Foster, Theresa; East of England Ambulance Service NHS Trust<br>Kirby, Kim; South Western Ambulance Service NHS Foundation Trust<br>Prothero, Larissa; East of England Ambulance Service NHS Trust<br>Purdy, Sarah; University of Bristol, Centre for Academic Primary Care<br>Solway, Chris; Alzheimer's Society<br>Benger, Jonathan; The University Hospitals NHS Foundation trust, Academic Department of Emergency care; The University of the West of England, Faculty of Health & Life Sciences

Keywords:	pre-hospital care, Emergency Ambulance Systems, Dementia < NEUROLOGY, GERIATRIC MEDICINE, Admission avoidance

SCHOLARONE™
Manuscripts

The use of emergency ambulance services by people with dementia: The HOMEWARD Project

Sarah Voss[1], Janet Brandling[1], Hazel Taylor[2], Sarah Black[3], Marina Buswell[4], Richard Cheston[1], Sarah Cullum[5], Theresa Foster[6], Kim Kirby[3], Larissa Prothero[6], Sarah Purdy[7], Chris Solway[8], Jonathan Benger[1,2]

1. University of the West of England, Faculty of Health and Applied Sciences, UK

2. Research Design Service, University Hospitals Bristol NHS Foundation Trust, UK

3. Research and Audit Department, South Western Ambulance Service NHS Foundation Trust, UK

4. Centre for Research in Public Health and Community Care, University of Hertfordshire, UK

5. Faculty of Medical and Health Sciences, University of Auckland, NZ

6. Research Support Services, East of England Ambulance Service NHS Trust, UK

7. School of Social and Community Medicine, University of Bristol, UK

8. Research Network, Alzheimer's Society, UK

Corresponding author:
Dr Sarah Voss, University of the West of England, Faculty of Health and Applied Sciences, Glenside Campus, Blackberry Hill, Bristol, BS16 1DD, UK. sarah.voss@uwe.ac.uk

Word count, excluding title page, abstract, references, figures and tables:
3270

Key words:
Pre-hospital care; Emergency Ambulance Systems; Dementia; Geriatrics, Admission avoidance

For peer review only

ABSTRACT (300 words)

Objectives

An increasing number of older people are calling ambulances and presenting to Accident and Emergency Departments. The presence of comorbidities and dementia can make managing these patients more challenging and hospital admission more likely, resulting in poorer outcomes for patients. However, we do not know how many of these patients are conveyed to hospital by ambulance, or if there is the opportunity to reduce unnecessary ambulance conveyance. This study aims to determine: how often ambulances are called to older people; how often comorbidities including dementia are recorded; the reason for the call; provisional diagnosis; the amount of time ambulance clinicians spend on scene; the frequency with which these patients are transported to hospital.

Methods

We conducted a retrospective observational cross-sectional study of ambulance Patient Care Records (PCRs) from calls to patients aged 65 years and over, in the geographical areas covered by two Ambulance Services in England: one in the east and one in the south west. Data was extracted from 3,037 PCRs with results reported as percentages and means with 95% confidence intervals.

Results

Dementia was recorded in 421 (13.9%) of PCRs. Patients with dementia were significantly less likely to be conveyed to hospital following a 999 call than those without dementia. The call cycle times were similar for patients regardless of whether or not they had dementia. Calls to people with dementia were more likely to be due to injury following a fall. In the overall sample, one or more comorbidities were reported on the PCR in over 80% of cases.

Conclusion

Rates of hospital conveyance for older people may be related to comorbidities, frailty and complex needs, rather than dementia. Further research is needed to understand the way in which ambulance clinicians make conveyance decisions at scene.

For peer review only - http://bmjopen.bmj.com/site/about/guidelines.xhtml

Strengths and limitations of this study

- This research examines the nature of ambulance service use by older people with dementia and comorbidities, and is the first study to describe this empirically.
- More than 3000 ambulance care records for patients aged over 65 years were examined. Data extraction was systematic using a pre-determined protocol.
- Data were collected on a number of key variables including presenting complaint, presence of dementia and other comorbidities, call cycle time and the frequency of conveyance.
- The study was retrospective and observational and carried out in two ambulance services in England; this may limit the generalisability of the findings.
- It was not possible to differentiate non-conveyance decisions that were made due to a patient's refusal to attend hospital.

For peer review only

For peer review only - http://bmjopen.bmj.com/site/about/guidelines.xhtml

Introduction

Dementia is a progressive and irreversible condition resulting in a decline of cognitive, functional, behavioural and psychological abilities, and an associated loss of independent living and social interaction. The number of people living with dementia is steadily increasing. In 2013, there were estimated to be 815,827 people with dementia in the UK; 773,502 were aged 65 years or over. This represents 1 in every 14 of the population aged 65 years and over.[1]

An increasing number of older people are accessing ambulance services and Accident and Emergency (A&E) departments.[2] The demands on urgent and emergency health care services are well documented and widespread.[3] Dementia is associated with higher levels of comorbidity than in an age matched population;[4,5] people with dementia have, on average, 2 to 8 additional chronic diseases.[6,7] As a result, dementia is associated with an increased risk of hospitalisation.[8] Notably, when comorbidities are adjusted for, people with dementia have a higher incidence of A&E attendance.[4] This means that where a patient's comorbidities include dementia, A&E attendance and hospital admission are all increased, compared to patients with the same comorbidities not including dementia.[8] The "conversion rate" (proportion of A&E attendances that become a hospital admission) is very high in this patient group.[9]

However, we do not know whether these patients who attend A&E and are subsequently admitted are conveyed to A&E by ambulance. There are approximately 8 million 999 ambulance calls and 20 million A&E attendances in the UK every year.[2] There is some anecdotal evidence that older people with dementia are more likely to be conveyed to hospital following an emergency ambulance call because ambulance clinicians cannot provide a full clinical assessment, or access alternative services that may be more suitable.[10] Reducing unnecessary conveyance to A&E could therefore lead to a reduction in both A&E utilisation and acute hospital admissions for older people with dementia.[9] This would be beneficial to patients and the wider health system. Patients with dementia tend to do poorly in acute hospital settings; cognitive impairment results in a reduced threshold for sensory overload and distress which can lead to disruptive behaviours and worse patient experience and outcomes, including high rates of discharge to care homes, readmission and death.[11-13] In addition, over 40% of unplanned admissions of those aged over 70 are for people living with dementia, [14] and it is estimated that 25% of hospital beds in the UK are occupied by people with dementia with significant consequences for patient flow and healthcare resources.[15]

For peer review only

The use of emergency ambulance services by older people with dementia is not well understood, and has been the subject of very little research. A review by Buswell et al [10] highlights the issue of 'inappropriate' calls, where an ambulance is called as the last resort or a "safety net". The authors identified recurrent themes including the absence of alternatives, and a lack of integration in healthcare. However, these assumptions have yet to be tested empirically.

Before exploring the opportunity to research interventions to reduce ambulance conveyance for this patient group, there is a need to determine the extent to which people with dementia, and other comorbidities, use ambulance services and are subsequently conveyed to hospital. Buswell et al studied the records of ambulance calls to people aged over 75 years and found that dementia was recorded in 14.5% of care records, with an additional 7.0% containing details suggestive of dementia or cognitive impairment.[16] Approximately 15% of this patient cohort resided in care homes.

The aim of this research was to determine how often ambulances are called to older people with comorbidities including dementia, and if these patients are more likely to be conveyed to hospital. We also sought to determine: the reason for the call; the provisional diagnosis; the amount of time ambulance clinicians spend on scene. Given the evidence available to date, we hypothesised that when a person with comorbidities including dementia required care, the clinicians would spend more time on scene, and patients would be more likely to be conveyed to hospital when compared to patients without dementia.

For peer review only - http://bmjopen.bmj.com/site/about/guidelines.xhtml

Methods

This was a retrospective observational cross-sectional study of ambulance Patient Care Records (PCRs) from calls to patients aged 65 years and over in the geographical areas covered by the West Division of South Western Ambulance Service NHS Foundation Trust (SWASFT) and East of England Ambulance Service NHS Trust (EEAST). Data were collected from each Trust during 24 hour periods in January 2017 and July 2017 (for EEAST) and 48 hour periods in the same months for SWASFT.

PCRs from calls to patients aged 65 years and over within the geographical area covered by SWASFT West Division were accessed via a bespoke query run on the Trust's electronic care system, and the anonymised data were sent to the research team. In EEAST, electronic versions of PCRs were securely stored and interrogated using computer software. Access to these records was overseen by the clinical audit teams for the respective Trusts.

The records were examined by two researchers using a standard template and the data recorded using a coding structure (Appendix A). The template and coding structure were determined by the study team in advance of data extraction by obtaining a pilot sample of 40 PCRs from a different time frame which were scrutinised for relevant data. This provided a guide for the researcher to obtain information on: the time of the call; the length of the call; the reason for the call; whether dementia and any other comorbidities were noted (supplementary material available on request); whether the patient lived alone, was in their own home or a care home; whether the patient was conveyed to hospital; any further referrals that were made. Electronic data were stored in a secure area of the University of the West of England and the University of Bristol servers. In order to assess inter-rater reliability, a sample of 64 PCRs from EEAST were extracted and coded by two additional members of the research team with agreement assessed using the Kappa statistic.

Percentages and means with 95% confidence intervals were used to report the key results. The analysis is exploratory, seeking to describe the nature of ambulance calls to patients with and without dementia, and therefore uses descriptive statistics only. Statistical hypothesis testing was not conducted due to the number of factors being considered and the risk of finding spurious statistically significant results. Moreover, as the clinical decisions made during call-outs are likely to be influenced by, for example, the crew, day of the week and time of the call, all of these unknown factors would need to be taken into account to make hypothesis testing valid.

For peer review only - http://bmjopen.bmj.com/site/about/guidelines.xhtml

Patient and public involvement:

This project was partnered by the Alzheimer's Society Research Network who were consulted on the draft protocol and gave feedback on the design, methodology, analysis and dissemination plans. They also provided specific and detailed advice on the proposed data fields. An experienced patient and public representative (CS) was a member of the study management group and co-author for this paper and contributed to all aspects of the project from inception to dissemination. Two additional PPI representatives were members of the Steering Committee.

For peer review only

For peer review only - http://bmjopen.bmj.com/site/about/guidelines.xhtml

Results

In total, 3,037 Patient Care Records (PCRs) were extracted: 2,311 from SWASFT and 726 from EEAST. The kappa statistic demonstrated good reliability, 26 of 37 items had a kappa of 0.61 or more indicating substantial agreement, including the coding of cognitive state (kappa = 0.683) and definite dementia diagnosis (kappa = 0.947).

Patient and call demographics

Dementia was recorded in the PCR in 421 cases (13.9%; 95% CI: 12.7%, 15.1%). The comparator group consisted of 2,567 (84.5%) cases where dementia was not recorded. 49 (1.6%) of PCRs were excluded because it was not clear whether dementia was recorded or not. See Table 1 for a summary of the patient demographics. The mean age of patients in the dementia group was 85.1 years (95% CI: 84.4, 85.7) and 80.7 years (95% CI: 80.4, 81.0) in the comparator group. Ambulance calls to people with dementia were more likely to be to a residential or nursing home (41.9%; 95% CI: 35.6%, 48.3%) than in the comparator group (5.4%; 95% CI: 4.3%, 6.7%), whereas calls to individuals living in their own home were more likely in the comparator group (81.0%; 95% CI: 78.8%, 83.0%) than the dementia group (52.8%; 95% CI: 46.4%, 59.2%).

Table 1: Summary of patient demographics (St. D = standard deviation; SWASFT = South Western Ambulance Service NHS Foundation Trust).

	**Comparator**		**Dementia**		**Total***	
	**N = 2,567**		**N= 421**		**N=3,037**	
	Mean	St. D	Mean	St. D	Mean	St. D
Age (years)	80.7	8.7	85.1	7.0	81.4	8.6
Number of ambulance staff on scene (SWASFT)	2.7	1.4	2.7	1.7	2.7	1.4
Gender	n	%	n	%	n	%
Male	1,162	45.4	166	39.5	1,347	44.5
Female	1,396	54.6	254	60.5	1,680	55.5
Location**	n = 1,390	%	N = 246	%	N = 1,665	%
Home	1,126	81.0%	130	52.8%	1,275	76.6%
Care home	75	5.4%	103	41.9%	186	11.2%
Public place	118	8.5%	7	2.9%	126	7.6%
Other	71	5.1%	6	2.4%	78	4.7%

* includes the 49 patients excluded due to uncertainty as to whether dementia was recorded.

** 1,372 missing

For peer review only - http://bmjopen.bmj.com/site/about/guidelines.xhtml

Presenting condition and provisional diagnosis

The reason for the call (presenting condition) was less likely to be related to a cardiac or respiratory problem in the dementia group (4.8%; 95% CI: 3.0%, 7.3% and 5.8%; 95% CI: 3.7%, 8.4%) than the comparator group (10.8%; 95% CI: 9.6%, 12.1% and 11.2%; 95% CI: 10.0%, 12.5%), but more likely to be for a fall in the dementia group (15.6%; 95% CI: 12.2%, 19.4%) than in the comparator group (9.1%; 95% CI: 8.0%, 10.3%) (Figure 1). Similar findings were evident for the provisional diagnosis as recorded by the attending clinician (Figure 2). Patients with dementia were less often diagnosed with cardiac or respiratory problems (4.6%; 95% CI: 2.8%, 7.1% and 5.4%; CI: 3.4%, 8.0%) than patients in the comparator group (11.7%; 95% CI: 10.4%, 13.0% and 11.1%; 95% CI: 9.9%, 12.4%). Conversely, traumatic injuries and falls were more prevalent in the dementia group (20.0%; 95% CI: 16.2%, 24.1% and 11.4%; 95% CI 8.5%, 14.9%) than in the comparator group (13.5%; 95% CI: 12.2%, 14.9% and 7.3%; 95% CI: 6.3%, 8.4%).

Figure 1: Provisional Diagnosis (N = 2,848: 411 with dementia and 2,437 comparator group)

Social circumstances and frailty

Patients with dementia were more likely to be living in a care home and to have a care package in place (Figure 2). Only 6.7% (95% CI: 4.2%, 10.0%) of patients with dementia were recorded as living in their own home without a care package compared to 31.7% (95% CI: 29.5%, 33.9%) of the comparator group. Similarly, 12.4% (95% CI: 9.0%, 16.6%) of patients with dementia were living with extended family without a care package compared to 32.1% (95% CI: 30.0%, 34.3%) of the comparator group. 25.8% (95% CI: 21.0%, 31.0%) and 22.6% (95% CI: 18.1%, 27.6%) of patients with dementia were living in a nursing or residential home compared with 3.5% (95% CI: 2.7%, 4.5%) and 3.6% (95% CI: 2.8%, 4.6%) of the comparator group. Frailty scores were recorded by SWASFT in 1,128 patients of which 70.2% (95% CI: 63.1%, 76.6%) of patients in the dementia group were assessed as moderately to severely frail compared to 30.5% (95% CI: 27.6%, 33.6%) in the comparator group. Only 7.4% (95% CI: 4.1%, 12.2%) of patients in the dementia group were assessed as managing well or being very fit compared to 43.7 (95% CI: 40.5%, 47.0%) in the comparator group (Table 2).

Figure 2: Living and social arrangements

For peer review only - http://bmjopen.bmj.com/site/about/guidelines.xhtml

Table 2: Frailty (N = 1128 with frailty data)

Frailty score	Comparator Group		Dementia Group	
	N	%	N	%
**Very Fit**	40	4.3	1	0.5
**Well**	137	14.6	2	1.1
**Managing well**	234	24.9	11	5.9
**Vulnerable**	111	11.8	18	9.6
**Mildly frail**	131	13.9	24	12.8
**Moderately frail**	147	15.6	43	22.9
**Severely Frail**	108	11.5	80	42.6
**Very severely frail**	15	1.6	6	3.2
**Terminally ill**	17	1.8	3	1.6
**Total**	940	100	188	100

Call cycle time and conveyance to hospital

The mean duration of the call (time of arrival on scene to time of closing the call) was shorter for people with dementia (85.2 minutes 95% CI: 81.5, 88.8) than for the comparator group (89.8 minutes 95% CI: 88.2, 91.5) (Table 3). In 61.0% of all cases the patient was conveyed to hospital by ambulance. Patients in the dementia group were less likely to be taken to hospital (50.4%); 95% CI: 45.5%, 55.2% compared to those in the comparator group (62.7; 95% CI: 60.8%, 64.6%). Fewer patients in the dementia group were recorded as experiencing pain (25.6%; 95% CI: 21.5%, 30.1%) than in the comparator group (39.5%; 95% CI: 37.6%, 41.4%).

Table 3: Call cycle time and conveyance to hospital (St. D = standard deviation; SWASFT = South Western Ambulance Service NHS Foundation Trust).

	Comparator		Dementia		Total	
	N = 2,567		N= 421		N=3,037	
	Mean	St D	Mean	St D	Mean	St D
Call cycle time in mins	89.8	41.3	85.2	38.3	89.4	40.9
	n	%	n	%	n	%
Number conveyed*	1,598/2,547	62.7	211/419	50.4	1,838/3,015	61.0
Number with Pain	1014	39.5	108	25.6	1132	37.3

For peer review only - http://bmjopen.bmj.com/site/about/guidelines.xhtml

Number with past medical history**	1,697/1,773	95.7	300/303	99.0	2,024/2,104	96.2

*22 missing

** SWAST only (207 missing)

Comorbidities

One or more comorbidities were recorded for 2,469 of the 3,037 (81.3%; 95% CI: 79.9%, 82.7%) cases. For patients in the dementia group, 352 of the 421 cases (83.6%; 95% CI: 79.7%, 87.0%) had at least one comorbidity recorded, and for the comparator group it was 2,076 of 2,567 cases (80.9%; 95% CI: 79.3%, 82.4%).

Respiratory and cardiac comorbidities were less likely to be recorded for patients with dementia, whereas neurological disorders, stroke and musculo-skeletal conditions were more prevalent (Figure 3). The presence of one or more comorbidities increased call cycle time and conveyance rate, irrespective of dementia (Table 3).

Figure 3: Comorbidities recorded

Table 3: Hospital conveyance and call cycle time with comorbidities

	**Conveyed to hospital**			**Call cycle time (minutes)**		
	**n**	**%**	**95% CI**	**n**	**mean**	**95% CI**
**Total**	1,838/3,015	61.0%	(59.2%, 62.7%)	2,996	89.4	(87.9, 90.9)
**Comparator**	1,598/2,547	62.7%	(60.8%, 64.6%)	2,532	89.8	(88.2, 91.5)
**Comparator (no comorbidities)**	273/481	56.8%	(52.2%, 61.2%)	480	82.8	(79.1, 86.6)
**Comparator (with comorbidities)**	1,325/2,066	64.1%	(62.0%, 66.2%)	2,052	91.5	(89.7, 93.3)
**Dementia**	211/419	50.4%	(45.5%, 55.2%)	418	85.2	(81.5, 88.8)
**Dementia (no comorbidities)**	33/69	47.8%	(35.6%, 60.2%)	69	78.7	(70.5, 86.9)
**Dementia (with comorbidities)**	178/350	50.9%	(45.5%, 56.2%)	349	86.4	(82.3, 90.5)

For peer review only - http://bmjopen.bmj.com/site/about/guidelines.xhtml

Discussion

Dementia was recorded in the Patient Care Record (PCR) in approximately 14% of cases. This finding is consistent with work carried out by Buswell et al,[16] which reported a rate of 14.5% in patients aged over 75 years.

Contrary to our hypotheses, patients aged over 65 years with dementia were less likely to be conveyed to hospital following a 999 call than patients over 65 years without dementia. In addition, the call cycle times were similar for patients regardless of whether or not they had dementia. This might indicate that the high A&E attendance rate for people with dementia observed in previous research [6,7] results from 'front door' attendances by people brought in by carers or relatives, rather than those conveyed to hospital by ambulance. Moreover, increasing awareness of the negative outcomes associated with hospital admission for people with dementia may lead to ambulance staff avoiding conveyance.

One or more comorbidities were reported in the PCR in just over 80% of all cases, with little difference between the dementia group and those without dementia. This is incongruent with findings from other settings, where people with dementia have consistently been found to have a significantly higher incidence of comorbidity at hospitalisation [4,8] and in primary care.[5] This may be related to the fact that information recorded in ambulance records is obtained from sources on scene and is not linked reliably to other records of health care data. Reliable and accurate information about past medical history is not always available to ambulance staff. Nonetheless, when one or more comorbidities were recorded as present, patients were more likely to be conveyed to hospital. This difference was more evident in the comparator group than in the dementia group. In addition, call cycle times were longer for patients with comorbidities, regardless of the presence of dementia. It may therefore be deduced that call cycles last longer and conveyance rates are higher when older adults have complex needs or frailty associated with comorbidity; the presence of dementia is not necessarily an influencing factor in these outcomes.

Post-hoc analyses of conveyance to hospital and call cycle time were calculated on patients in different age bands (65-74, 75-84 and 85+ years). The findings indicate that in general, for younger people the call cycle time is longer and they are more likely to be conveyed to hospital. This is consistent with previous research on the epidemiology of non-conveyed patients, which found that older patients account for the majority of these calls. Moreover, falls account for 34%-40% of all non-conveyed patients.[17,18]

For peer review only - http://bmjopen.bmj.com/site/about/guidelines.xhtml

Dementia is associated with an increased risk of falls, and an increased level of fall related traumatic injury (31% vs 21%) was noted for this patient group. In previous research, Buswell et al [16] found falls to be the most common reason for a 999 call; in the present study, while a fall was the presenting complaint in over a quarter of cases overall, this proportion increased to almost half where dementia was recorded. Therefore, it is possible that the high number of falls in the dementia group accounts for the lower rate of conveyance. It was not possible in this research to establish which non-conveyance decisions were made by the attending clinician, and which were due to the patient's refusal to be transported, and this may also be a relevant factor. However, non-conveyance in older people who have fallen may be problematic since those not conveyed have been found to have a high rate of subsequent emergency healthcare contacts and an increased risk of death and hospital admission [18]. Although there is research ongoing to address prehospital assessment and management of people who have fallen [19], it is important to consider the impact of dementia in these protocols.

Of particular relevance to older people who have dementia, who have fallen and who are not conveyed is the assessment of pain; it is well recognised that pain is less often appreciated in dementia patients and is difficult to assess.[20,21] Despite the fact that we observed falls to be more common in the dementia group, pain was less often recorded in PCRs for those with dementia (25.6%) than for those in the comparator group (39.5%). More research is needed to establish reliable ways of assessing pain in people with dementia, to ensure that non-conveyance decisions are appropriate and do not lead to a further need for emergency healthcare.

Unsurprisingly, patients with dementia were more likely to be living in a care home (48%) than those without dementia (7%). Conveyance was more frequent for both groups when living in a care home than for those living in their own home; post-hoc analysis indicated that when in a care home, people with dementia are still less likely to be conveyed to hospital (54.6%) than those without (66.9%). Therefore, it is possible that care home staff do not always feel able to accurately assess these patients, and may rely on ambulance staff who, in turn, need to be equipped and skilled to reliably identify pain and assess other symptoms in people with dementia.

There are several limitations that impact on the generalisability of the findings and the conclusions that can be drawn from our study. Firstly, this was a retrospective observational study and there is

For peer review only - http://bmjopen.bmj.com/site/about/guidelines.xhtml

the potential for bias that may affect the selection of comparator cases. However, great care was taken to ensure data extraction was robust and this is detailed in the coding template. Secondly, the samples were only extracted from two services over two short periods. The time periods were in January and July to capture any seasonal variation. Additional issues that may have affected the reliability and validity of the data were associated with dementia diagnosis; recording of dementia on the PCR does not equate to a definitive diagnosis. Conversely, it is likely that some of the comparator cases had undiagnosed or unrecognised dementia. Nonetheless, in the absence of robust evidence in this field, this study provides both useful information and indications for further research.

Conclusion

The reason for a lower conveyance rate in patients recorded as having dementia remains unexplained. In this study comorbidity was a more accurate predictor of conveyance to hospital than dementia per se. We have, however, shown that ambulance services are called to people with dementia for different reasons than people without dementia. Therefore, it is possible that the lower conveyance rate for dementia patients may be associated with non-injury falls or transient symptoms that are not perceived as requiring admission to hospital. However, unless pain can be reliably assessed, decisions on non-conveyance for people with dementia may not necessarily be appropriate. In addition, care and nursing staff may have low thresholds for calling an ambulance for an individual that requires primary care management rather than conveyance to hospital. This would offer an explanation for the similar call cycle times we observed for both patients groups. However, further research is required to examine these issues in more detail.

This study has shown that higher rates of hospital admission for older people may be more related to complex needs and frailty than dementia. Given the unexpected nature of our findings, further research is required to understand how people with dementia interact with ambulance services, and the way in which ambulance clinicians make decisions at scene. There is much evidence to suggest that hospital admission for people with dementia results in adverse patient outcomes, and should be avoided where possible. However, evidence also suggests that patients who are not conveyed following a fall are at risk of poorer outcomes. Research on the assessment of pain and injury following a fall in people with dementia is a high priority, to ensure the most appropriate conveyance decisions are made.

For peer review only - http://bmjopen.bmj.com/site/about/guidelines.xhtml

Acknowledgments:

The authors would like to thank members of the Study Steering Committee for their contribution to this research: Professor Steve Iliffe (Professor of Primary Care for Older People, University College London); Professor Philp Bath (The Stroke Association Professor of Stroke Medicine, Chair and Head of the Division of Clinical Neuroscience and NIHR Senior Investigator, The University of Nottingham), Elinor Griffiths (Research Grants Manager, University Hospitals Bristol NHS Foundation Trust); Simon Goodwin (Programme Manager, Central Commissioning Facility, NIHR); Alan Bradley and John Long (Patient and Public advisors).

Funding statement:

This report is independent research funded by the National Institute for Health Research (Research for Patient Benefit Programme, Home or hospital for people with dementia and one or more other multimorbidities: what is the potential to reduce avoidable emergency admissions?, PB-PG-0215-36098). The views expressed in this publication are those of the author(s) and not necessarily those of the NHS, the National Institute for Health Research or the Department of Health.

Author contributions:

SV, JB, SB, MB, RC, SC, KK, SP and CS were involved in the conception and design of the study. SV, JB, JBr, SB, MB, RC, SC, TF, KK, LP, SP and CS made substantial contributions to development of the methodology and analysis. SV drafted the manuscript. JBr, TF, KK and LP led on the data collection and HT conducted the data analysis. All authors contributed to the critical revision of the manuscript for publication and approved the final version to be published.

Competing interests:

All authors have completed the ICMJE uniform disclosure form at www.icmje.org/coi_disclosure.pdf and declare: no support from any organisation for the submitted work; no financial relationships with any organisations that might have an interest in the submitted work in the previous three years; no other relationships or activities that could appear to have influenced the submitted work.

Data sharing statement:

For peer review only - http://bmjopen.bmj.com/site/about/guidelines.xhtml

Supplementary files and the dataset are available from sarah.voss@uwe.ac.uk.

For peer review only

REFERENCES

1. Alzheimer's Society (2014) https://www.alzheimers.org.uk/info/20027/news_and_media/541/facts_for_the_media (Accessed 17/01/2018)
2. NHS Digital (2016). Hospital Admitted Patient Care Activity, 2015-16. http://digital.nhs.uk/catalogue/PUB22378 (Accessed 17/01/2018)
3. Kings Fund (2017) Quarterly Monitoring Report (QMR) 22. http://qmr.kingsfund.org.uk/2017/22/ (Accessed 17/01/2018)
4. Zhao Y, Kuo T, Weir S, et al. Healthcare costs and utilization for Medicare beneficiaries with Alzheimer's. *BMC Health Serv Res* 2008;8(1):108.
5. Poblador-Plou B, Calderón-Larrañaga A, Marta-Moreno J, et al. Comorbidity of dementia: a cross-sectional study of primary care older patients. *BMC psychiatry* 2014;14(1):84.
6. Sanderson M, Wang J, Davis D, et al. Co-morbidity associated with dementia. *Am J Alzheimers Dis Other Demen* 2002;17(2):73-78.
7. Schubert C, Boustani M, Callahan C, et al. Comorbidity profile of dementia patients in primary care: are they sicker? *J Am Geriatr Soc* 2006;54(1):104-109.
8. Phelan A, Borson S, Grothaus L, et al. Association of incident dementia with hospitalizations. *Jama* 2012;307(2):165-172.
9. Wittenberg R, Sharpin L, McCormick B, et al. Understanding Emergency Hospital Admissions of Older People. Centre for Health Service Economics and Organisation. Report 6, December 2014. See: https://www.chseo.org.uk/downloads/report6-emergencyadmissions.pdf
10. Buswell M, Lumbard P, Prothero L, et al. Unplanned, urgent and emergency care: what are the roles that EMS plays in providing for older people with dementia? An integrative review of policy, professional recommendations and evidence. *Emerg Med J*. 2014 Dec 2:emermed-2014.. Emergency Medicine Journal, emermed-2014.
11. Ferns T. Violence, aggression and physical assault in healthcare settings. *Nursing standard*, 2006;21(13):42-46.
12. Hodge A, Marshall A. Violence and aggression in the emergency department: A critical care perspective. *Australian Critical Care* 2007;20(2):61-67.
13. Gitlin L, Winter L, Burke J. Tailored activities to manage neuropsychiatric behaviors in persons with dementia and reduce caregiver burden: a randomized pilot study. *Am J Geriatr Psychiatry* 2008;16(3):229-239.
14. Sampson EL, Blanchard MR, Jones L, Tookman A, King M. Dementia in the acute hospital: prospective cohort study of prevalence and mortality. The British Journal of Psychiatry. 2009 Jul 1;195(1):61-6.
15. Alzheimer's Society. Counting the Cost: Caring for people with dementia on hospital wards. Alzheimer's Society; 2009.
16. Buswell M, Lumbard P, Fleming J, et al. Using ambulance service PCRs to understand 999 call-outs to older people with dementia. *Journal of Paramedic Practice* 2016;8(5):246-251.
17. Marks PJ, Daniel TD, Afolabi O, Spiers G, Nguyen-Van-Tam JS. Emergency (999) calls to the ambulance service that do not result in the patient being transported to hospital: an epidemiological study. Emergency Medicine Journal. 2002 Sep 1;19(5):449-52.
18. Snooks HA, Halter M, Close JC, Cheung WY, Moore F, Roberts SE. Emergency care of older people who fall: a missed opportunity. BMJ Quality & Safety. 2006 Dec 1;15(6):390-2.

For peer review only - http://bmjopen.bmj.com/site/about/guidelines.xhtml

19. Snooks H, Anthony R, Chatters R, Cheung WY, Dale J, Donohoe R, Gaze S, Halter M, Koniotou M, Logan P, Lyons R. Support and assessment for fall emergency referrals (SAFER 2) research protocol: cluster randomised trial of the clinical and cost effectiveness of new protocols for emergency ambulance paramedics to assess and refer to appropriate community-based care. BMJ open. 2012 Jan 1;2(6):e002169.
20. Lord B. Paramedic assessment of pain in the cognitively impaired adult patient. *BMC Emerg Med* 2009;9(1):20.
21. Care Quality Commission. Cracks in the Pathway. People's Experiences of Dementia Care as They Move between Care Homes and Hospitals. Newcastle upon Tyne. 2014;Care Quality Commission.

For peer review only

For peer review only - http://bmjopen.bmj.com/site/about/guidelines.xhtml

Figure 1: Provisional Diagnosis (N = 2848, N = 411 with dementia and N = 2437 for comparator)

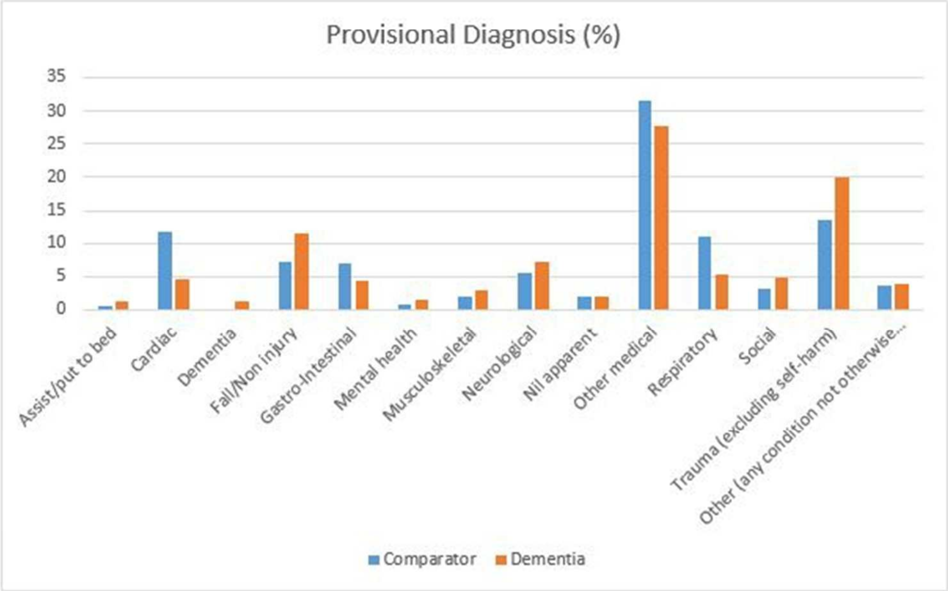

168x118mm (96 x 96 DPI)

Figure 2: Living social arrangements

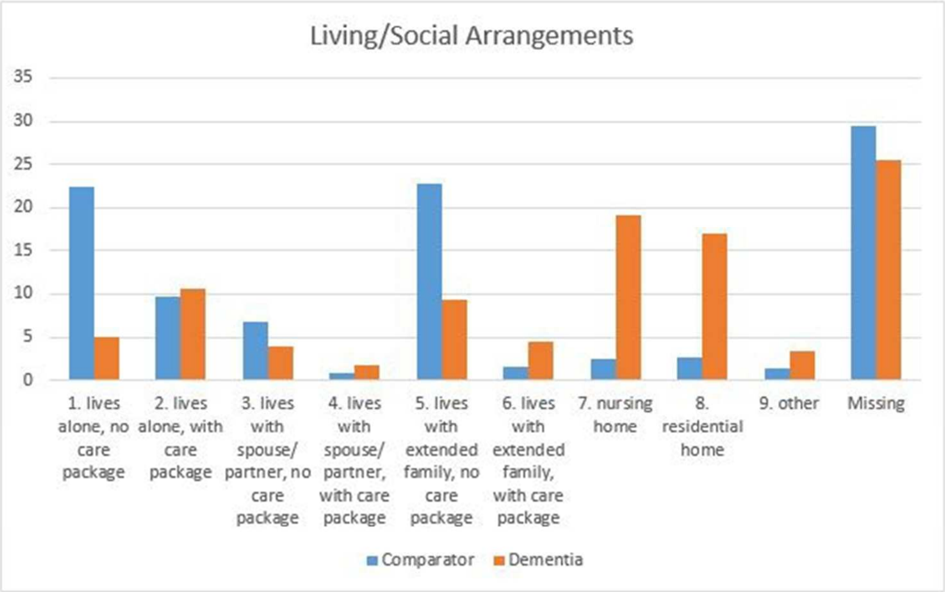

Note: data missing for 29.5% of comparator group and 25.4% of dementia group

169x134mm (96 x 96 DPI)

Figure 3: Comorbidities recorded

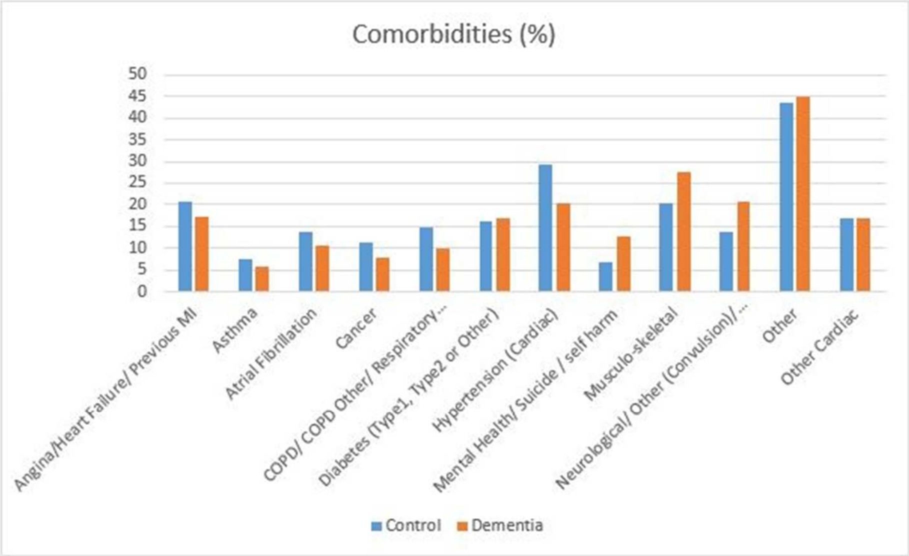

175x130mm (96 x 96 DPI)

Appendix A: HOMEWARD Phase 1- Coding for SWASFT/ EEAST Data

CODES

Call Colour codes:

1- red
2- green
3- amber- not available in EEAST

See Treat or Convey codes

1- See & Treat
2- See & Convey (include transport only in EEAST)

Qualification codes- identify most senior clinician recorded at scene

1. Technician
2. Paramedic/SWAMP paramedic
3. Lead Paramedic
4. Specialist Paramedic/Critical Care Paramedic
5. Other- contains the following
    a. BLS trained (first responder)
    b. Ambulance Care Assistant
    c. Emergency Care Assistant
    d. Student Paramedic
    e. Operational Officer
    f. Doctor

Gender codes

1- Male
2- Female

Presenting condition codes

SWASFT	EEAST
1- General	Other
2- Cardiac	AF, Heart Failure, Hypertension, MI, other cardiac
3- Respiratory	Asthma, COPD, other respiratory
4- Endocrine	Diabetes I or II, also other for thyroid
5- Neurological	Stroke/ CVA, TIA, Other neurological
6- Gastro-Intestinal	Other
7- Musculoskeletal	Musculoskeletal
8- Obs/gynae	Other
9- Cancer	Other
10- Mental health	Mental Health
11- Trauma (excluding self-harm)	Trauma
12- Other medical	Other
13- Social	Social
14- Other (any condition not otherwise specified)	Other

For peer review only - http://bmjopen.bmj.com/site/about/guidelines.xhtml

15- Deliberate self-harm	Suicide/ self-harm
16- Environmental	Other
(17- Sepsis) see other medical	Other e.g. UTI, Chest Infection
18- Poisoning (accidental)	Other
19- Burns	Other
	Cognitive impairment/ confusion- delirium
	Dementia
	Nil apparent
	Palliative

Provisional diagnosis codes

SWASFT	EEAST
1- General	Other
2- Cardiac	AF, Heart Failure, Hypertension, MI, other cardiac
3- Respiratory	Asthma, COPD, other respiratory
4- Endocrine	Diabetes I or II, also other for thyroid
5- Neurological	Stroke/ CVA, TIA, Other neurological
6- Gastro-Intestinal	Other
7- Musculoskeletal	Musculoskeletal
8- Obs/gynae	Other
9- Cancer	Other
10- Mental health	Mental Health
11- Trauma (excluding self-harm)	Trauma
12- Other medical	Other
13- Social	Social
14- Other (any condition not otherwise specified)	Other
15- Deliberate self-harm	Suicide/ self-harm
16- Environmental	Other
(17- Sepsis) see other medical	Other e.g. UTI, Chest Infection
18- Poisoning (accidental)	Other
19- Burns	Other
	Cognitive impairment/ confusion- delirium
	Dementia
	Nil apparent
	Palliative

For peer review only

Search terms for target and control groups:

Target group:

Dementia, Alzheimer's, Fronto-temporal dementia, vascular dementia, senility, Pick's disease, Lewy Body disease/ dementia

1- Dementia present - Presence of the diagnosis of one of the above
2- Dementia suspected - paramedic suggests dementia may be present eg ? dementia, seeing memory nurse, taking dementia medication
3- Dementia inferred - Paramedic infers ongoing problem through use of other language such mild cognitive impairment, cognitive impairment, memory loss. Sometimes this might also be called confusion but is <u>not</u> acute. This might be confirmed as 'normal or usual for the patient'.

Control group:

Confusion- acute, delirium,

4- Presence of one of the above

Drugs search terms:

Drugs: Acumor (XL), **Aricept**, Axurer, Consium XL, **Donepezil,** Ebixa, Elmino, Exelon, **Galantamine,** Galsya (XL), Gazylan XL, Gatalin (XL), Kerstipon, Lotprosin XL, Luventa XL, Maruxa, **Memantine,** Nemdatine, Nimvastid, Reminyl (XL), **Rivastigmine**, Valios.

PATCHES: Alzest, Eluden, Prometax, Rivatev, Voleze

1- Presence of these drugs in case record

Social History code:

1- lives alone, no care package
2- lives alone, with care package
3- lives with spouse/ partner, no care package
4- lives with spouse/ partner, with care package
5- lives with extended family, no care package
6- lives with extended family, with care package
7- nursing home
8- residential home
9- other

Location codes:

1- Home
2- Nursing home
3- Residential Home
4- Public place
5- Other
6- Not documented

For peer review only - http://bmjopen.bmj.com/site/about/guidelines.xhtml

Capacity to consent

1- Patient has capacity
2- Patient does not have capacity

Rockwood Frailty code:

1- Very Fit
2- Well
3- 3 Managing Well
4- Vulnerable
5- Mildly Frail
6- Moderately Frail
7- Severely Frail
8- Very Severely Frail
9- Terminally Ill

Falls

Search conducted in presenting condition and HPC for fall related calls. This is to ensure those coded later as trauma are also coded for falls. Falls are coded as present if they occurred within last 6 days and have contributed to the call.

Search terms fall, fell, slip, trip

For peer review only - http://bmjopen.bmj.com/site/about/guidelines.xhtml

STROBE Statement—Checklist of items that should be included in reports of ***cross-sectional studies***

	Item No	Recommendation	Page Number
**Title and abstract**	1	(*a*) Indicate the study's design with a commonly used term in the title or the abstract	1
		(*b*) Provide in the abstract an informative and balanced summary of what was done and what was found	2
**Introduction**			
Background/rationale	2	Explain the scientific background and rationale for the investigation being reported	5
Objectives	3	State specific objectives, including any prespecified hypotheses	5
**Methods**			
Study design	4	Present key elements of study design early in the paper	6
Setting	5	Describe the setting, locations, and relevant dates, including periods of recruitment, exposure, follow-up, and data collection	6
Participants	6	(*a*) Give the eligibility criteria, and the sources and methods of selection of participants	6
Variables	7	Clearly define all outcomes, exposures, predictors, potential confounders, and effect modifiers. Give diagnostic criteria, if applicable	6
Data sources/ measurement	8*	For each variable of interest, give sources of data and details of methods of assessment (measurement). Describe comparability of assessment methods if there is more than one group	*6*
Bias	9	Describe any efforts to address potential sources of bias	6
Study size	10	Explain how the study size was arrived at	6
Quantitative variables	11	Explain how quantitative variables were handled in the analyses. If applicable, describe which groupings were chosen and why	6
Statistical methods	12	(*a*) Describe all statistical methods, including those used to control for confounding	6
		(*b*) Describe any methods used to examine subgroups and interactions	6
		(*c*) Explain how missing data were addressed	6
		(*d*) If applicable, describe analytical methods taking account of sampling strategy	6
		(*e*) Describe any sensitivity analyses	6
**Results**			
Participants	13*	(a) Report numbers of individuals at each stage of study—eg numbers potentially eligible, examined for eligibility, confirmed eligible, included in the study, completing follow-up, and analysed	8-11
		(b) Give reasons for non-participation at each stage	8-11
		(c) Consider use of a flow diagram	N/A
Descriptive data	14*	(a) Give characteristics of study participants (eg demographic, clinical, social) and information on exposures and potential confounders	8-11
		(b) Indicate number of participants with missing data for each variable of interest	8-11
Outcome data	15*	Report numbers of outcome events or summary measures	8-11
Main results	16	(*a*) Give unadjusted estimates and, if applicable, confounder-adjusted estimates and their precision (eg, 95% confidence interval). Make clear which confounders were adjusted for and why they were included	8-11

For peer review only

		(*b*) Report category boundaries when continuous variables were categorized	8-11
		(*c*) If relevant, consider translating estimates of relative risk into absolute risk for a meaningful time period	8-11
Other analyses	17	Report other analyses done—eg analyses of subgroups and interactions, and sensitivity analyses	8-11
**Discussion**			
Key results	18	Summarise key results with reference to study objectives	12
Limitations	19	Discuss limitations of the study, taking into account sources of potential bias or imprecision. Discuss both direction and magnitude of any potential bias	13-14
Interpretation	20	Give a cautious overall interpretation of results considering objectives, limitations, multiplicity of analyses, results from similar studies, and other relevant evidence	12-14
Generalisability	21	Discuss the generalisability (external validity) of the study results	14
**Other information**			
Funding	22	Give the source of funding and the role of the funders for the present study and, if applicable, for the original study on which the present article is based	15

*Give information separately for exposed and unexposed groups.

Note: An Explanation and Elaboration article discusses each checklist item and gives methodological background and published examples of transparent reporting. The STROBE checklist is best used in conjunction with this article (freely available on the Web sites of PLoS Medicine at http://www.plosmedicine.org/, Annals of Internal Medicine at http://www.annals.org/, and Epidemiology at http://www.epidem.com/). Information on the STROBE Initiative is available at www.strobe-statement.org.

For peer review only

For peer review only - http://bmjopen.bmj.com/site/about/guidelines.xhtml

BMJ Open

How do people with dementia use the ambulance service? A retrospective study in England: The HOMEWARD Project

Journal:	*BMJ Open*
Manuscript ID	bmjopen-2018-022549.R1
Article Type:	Research
Date Submitted by the Author:	14-May-2018
Complete List of Authors:	Voss, Sarah; University of the West of England, Health and Life Sciences<br>Brandling, Janet; University of the West of England, Health and Life Sciences<br>Taylor, Hazel; Research Design Service South West,<br>Black, Sarah; South Western Ambulance Service NHS Foundation Trust,<br>Buswell, Marina; University of Hertfordshire, CRIPACC< School of Health and Social Work<br>Cheston, Richard; University of the West of England, Health and Social Sciences<br>Cullum, Sarah; University of Auckland<br>Foster, Theresa; East of England Ambulance Service NHS Trust<br>Kirby, Kim; South Western Ambulance Service NHS Foundation Trust<br>Prothero, Larissa; East of England Ambulance Service NHS Trust<br>Purdy, Sarah; University of Bristol, Centre for Academic Primary Care<br>Solway, Chris; Alzheimer's Society<br>Benger, Jonathan; The University Hospitals NHS Foundation trust, Academic Department of Emergency care; The University of the West of England, Faculty of Health & Life Sciences
**Primary Subject Heading**:	Emergency medicine
Secondary Subject Heading:	Mental health
Keywords:	pre-hospital care, Emergency Ambulance Systems, Dementia < NEUROLOGY, GERIATRIC MEDICINE, Admission avoidance

SCHOLARONE™ Manuscripts

How do people with dementia use the ambulance service? A retrospective study in England: The HOMEWARD Project

Sarah Voss[1], Janet Brandling[1], Hazel Taylor[2], Sarah Black[3], Marina Buswell[4], Richard Cheston[1], Sarah Cullum[5], Theresa Foster[6], Kim Kirby[3], Larissa Prothero[6], Sarah Purdy[7], Chris Solway[8], Jonathan Benger[1,2]

1. University of the West of England, Faculty of Health and Applied Sciences, UK

2. Research Design Service, University Hospitals Bristol NHS Foundation Trust, UK

3. Research and Audit Department, South Western Ambulance Service NHS Foundation Trust, UK

4. Centre for Research in Public Health and Community Care, University of Hertfordshire, UK

5. Faculty of Medical and Health Sciences, University of Auckland, NZ

6. Research Support Services, East of England Ambulance Service NHS Trust, UK

7. School of Social and Community Medicine, University of Bristol, UK

8. Research Network, Alzheimer's Society, UK

Corresponding author:
Dr Sarah Voss, University of the West of England, Faculty of Health and Applied Sciences, Glenside Campus, Blackberry Hill, Bristol, BS16 1DD, UK. sarah.voss@uwe.ac.uk

Word count, excluding title page, abstract, references, figures and tables:
2948

Key words:
Pre-hospital care; Emergency Ambulance Systems; Dementia; Geriatrics, Admission avoidance

For peer review only

ABSTRACT (300 words)

Objectives

An increasing number of older people are calling ambulances and presenting to Accident and Emergency Departments. The presence of comorbidities and dementia can make managing these patients more challenging and hospital admission more likely, resulting in poorer outcomes for patients. However, we do not know how many of these patients are conveyed to hospital by ambulance. This study aims to determine: how often ambulances are called to older people; how often comorbidities including dementia are recorded; the reason for the call; provisional diagnosis; the amount of time ambulance clinicians spend on scene; the frequency with which these patients are transported to hospital.

Methods

We conducted a retrospective cross-sectional study of ambulance Patient Care Records (PCRs) from calls to patients aged 65 years and over. Data were collected from two Ambulance Services in England during 24 or 48 hour periods in January 2017 and July 2017. The records were examined by two researchers using a standard template and the data were extracted from 3,037 PCRs using a coding structure.

Results

Results were reported as percentages and means with 95% confidence intervals. Dementia was recorded in 421 (13.9%) of PCRs. Patients with dementia were significantly less likely to be conveyed to hospital following an emergency call than those without dementia. The call cycle times were similar for patients regardless of whether or not they had dementia. Calls to people with dementia were more likely to be due to injury following a fall. In the overall sample, one or more comorbidities were reported on the PCR in over 80% of cases.

Conclusion

Rates of hospital conveyance for older people may be related to comorbidities, frailty and complex needs, rather than dementia. Further research is needed to understand the way in which ambulance clinicians make conveyance decisions at scene.

For peer review only - http://bmjopen.bmj.com/site/about/guidelines.xhtml

Strengths and limitations of this study

- This research examines the nature of ambulance service use by older people with dementia and comorbidities, and is the first study to describe this empirically.
- More than 3000 ambulance care records for patients aged over 65 years were examined. Data extraction was systematic using a pre-determined protocol.
- Data were collected on a number of key variables including presenting complaint, presence of dementia and other comorbidities, call cycle time and the frequency of conveyance.
- The study was retrospective and carried out in two ambulance services in England; this may limit the generalisability of the findings.
- It was not possible to differentiate non-conveyance decisions that were made due to a patient's refusal to attend hospital.

For peer review only

For peer review only - http://bmjopen.bmj.com/site/about/guidelines.xhtml

Introduction

Dementia is a progressive and irreversible condition resulting in a decline of cognitive, functional, behavioural and psychological abilities, and an associated loss of independent living and social interaction. The number of people living with dementia is steadily increasing. In 2013, there were estimated to be 815,827 people with dementia in the UK; 773,502 were aged 65 years or over. This represents 1 in every 14 of the population aged 65 years and over.[1]

An increasing number of older people are accessing ambulance services and Accident and Emergency (A&E) departments.[2] The demands on urgent and emergency health care services are well documented and widespread.[3] Dementia is associated with higher levels of comorbidity than in an age matched population;[4,5] More than 90% of people living with dementia have another health condition.[6] As a result, dementia is associated with an increased risk of hospitalisation.[7,8] Notably, when comorbidities are adjusted for, people with dementia have a higher incidence of A&E attendance.[4] This means that where a patient's comorbidities include dementia, A&E attendance and hospital admission are all increased, compared to patients with the same comorbidities not including dementia.[8] The "conversion rate" (proportion of A&E attendances that become a hospital admission) is very high in this patient group.[9]

However, we do not know whether these patients who attend A&E and are subsequently admitted are conveyed to A&E by ambulance. There are approximately 8 million emergency ambulance calls and 20 million A&E attendances in the UK every year.[2] There is some anecdotal evidence that older people with dementia are more likely to be conveyed to hospital following an emergency ambulance call because ambulance clinicians cannot provide a full clinical assessment, or access alternative services that may be more suitable.[10] Reducing unnecessary conveyance to A&E could therefore lead to a reduction in both A&E utilisation and acute hospital admissions for older people with dementia.[9] This would be beneficial to patients and the wider health system. Patients with dementia tend to do poorly in acute hospital settings; cognitive impairment results in a reduced threshold for sensory overload and distress which can lead to disruptive behaviours and worse patient experience and outcomes, including high rates of discharge to care homes, readmission and death.[11-13] In addition, over 40% of unplanned admissions of those aged over 70 are for people living with dementia, [14] and it is estimated that 25% of hospital beds in the UK are occupied by people with dementia with significant consequences for patient flow and healthcare resources.[15]

For peer review only - http://bmjopen.bmj.com/site/about/guidelines.xhtml

The use of emergency ambulance services by older people with dementia is not well understood, and has been the subject of very little research. A review by Buswell et al [10] highlights the issue of 'inappropriate' calls, where an ambulance is called as the last resort or a "safety net". The authors identified recurrent themes including the absence of alternatives, and a lack of integration in healthcare. However, these assumptions have yet to be tested empirically.

Before exploring the opportunity to research interventions to reduce ambulance conveyance for this patient group, there is a need to determine the extent to which people with dementia, and other comorbidities, use ambulance services and are subsequently conveyed to hospital. Buswell et al studied the records of ambulance calls to people aged over 75 years and found that dementia was recorded in 14.5% of care records, with an additional 7.0% containing details suggestive of dementia or cognitive impairment.[16] Approximately 15% of this patient cohort resided in care homes.

The aim of this research was to determine how often ambulances are called to older people with comorbidities including dementia, and if these patients are more likely to be conveyed to hospital. We also sought to determine: the reason for the call; the provisional diagnosis; the amount of time ambulance clinicians spend on scene. Given the evidence available to date, we hypothesised that when a person with comorbidities including dementia required care, the clinicians would spend more time on scene, and patients would be more likely to be conveyed to hospital when compared to patients without dementia.

For peer review only - http://bmjopen.bmj.com/site/about/guidelines.xhtml

Methods

This was a retrospective cross-sectional study of ambulance Patient Care Records (PCRs) from calls to patients aged 65 years and over in the geographical areas covered by the West Division of South Western Ambulance Service NHS Foundation Trust (SWASFT) and East of England Ambulance Service NHS Trust (EEAST). Data were collected from each Trust during 24 hour periods in January 2017 and July 2017 (for EEAST) and 48 hour periods in the same months for SWASFT.

PCRs from calls to patients aged 65 years and over within the geographical area covered by SWASFT West Division were accessed via a bespoke query run on the Trust's electronic care system, and the anonymised data were sent to the research team. In EEAST, electronic versions of PCRs were securely stored and interrogated using computer software. Access to these records was overseen by the clinical audit teams for the respective Trusts.

HRA approval (IRAS: 202449) was obtained prior to data collection. Approval from an ethics committee was not required because the project involved the collection and analysis of retrospective and anonymised patient data only.

The records were examined by two researchers using a standard template and the data recorded using a coding structure (Appendix A). The template and coding structure were determined by the study team in advance of data extraction by obtaining a pilot sample of 40 PCRs from a different time frame which were scrutinised for relevant data. This provided a guide for the researcher to obtain information on: the time of the call; the length of the call; the reason for the call; whether dementia and any other comorbidities were noted in any section or free text area of the PCR (supplementary material available on request); whether the patient lived alone, was in their own home or a care home; whether the patient was conveyed to hospital; any further referrals that were made. Electronic data were stored in a secure area of the University of the West of England and the University of Bristol servers. In order to assess inter-rater reliability, a sample of 64 PCRs from EEAST were extracted and coded by two additional members of the research team with agreement assessed using the Kappa statistic.

As the study uses retrospective data from ambulance Patient Care Records (PCRs), for some items the completion rates were low. However, the key items such as whether conveyance occurred and the length of call out had good completion rates. For most items the assumption was made that the data were "missing at random", and so those with missing data for the item in question were

For peer review only - http://bmjopen.bmj.com/site/about/guidelines.xhtml

removed from the analysis of that item only. For this reason the number in the analysis (n) is different for each item reported. For the comorbidities, it was assumed that if the comorbidity had not been reported, then the patient did not have it, since there was no option to record "No" for the individual comorbidities in the PCR.

Percentages and means with 95% confidence intervals were determined using recognised methods for calculating an estimate of the population standard deviation and used to report the key results. 95% confidence intervals for percentages/proportions were calculated using the Exact Binomial Method. 95% confidence intervals for means were calculated using standard methods assuming a normal distribution, which is appropriate given the large sample size. The analysis is exploratory, seeking to describe the nature of ambulance calls to patients with and without dementia, and therefore uses descriptive statistics only. Statistical hypothesis testing was not conducted due to the number of factors being considered and the risk of finding spurious statistically significant results. Moreover, as the clinical decisions made during call-outs are likely to be influenced by, for example, the crew, day of the week and time of the call, all of these unknown factors would need to be taken into account to make hypothesis testing valid.

Patient and public involvement:

This project was partnered by the Alzheimer's Society Research Network who were consulted on the draft protocol and gave feedback on the design, methodology, analysis and dissemination plans. They also provided specific and detailed advice on the proposed data fields. An experienced patient and public representative (CS) was a member of the study management group and co-author for this paper and contributed to all aspects of the project from inception to dissemination. Two additional PPI representatives were members of the Steering Committee.

For peer review only

For peer review only - http://bmjopen.bmj.com/site/about/guidelines.xhtml

Results

In total, 3,037 Patient Care Records (PCRs) were extracted: 2,311 from SWASFT and 726 from EEAST. The kappa statistic demonstrated good reliability, 26 of 37 items had a kappa of 0.61 or more indicating substantial agreement, including the coding of cognitive state (kappa = 0.683) and definite dementia diagnosis (kappa = 0.947).

Patient and call demographics

Dementia was recorded in the PCR in 421 cases (13.9%; 95% CI: 12.7%, 15.1%). The comparator group consisted of 2,567 (84.5%) cases where dementia was not recorded. 49 (1.6%) of PCRs were excluded because it was not clear whether dementia was recorded or not. See Table 1 for a summary of the patient demographics. The mean age of patients in the dementia group was 85.1 years (95% CI: 84.4, 85.7) and 80.7 years (95% CI: 80.4, 81.0) in the comparator group. Ambulance calls to people with dementia were more likely to be to a residential or nursing home (41.9%; 95% CI: 35.6%, 48.3%) than in the comparator group (5.4%; 95% CI: 4.3%, 6.7%), whereas calls to individuals living in their own home were more likely in the comparator group (81.0%; 95% CI: 78.8%, 83.0%) than the dementia group (52.8%; 95% CI: 46.4%, 59.2%).

Table 1: Summary of patient demographics (St. D = standard deviation; SWASFT = South Western Ambulance Service NHS Foundation Trust).

	**Comparator**		**Dementia**		**Total***	
	**N = 2,567**		**N= 421**		**N=3,037**	
	Mean	St. D	Mean	St. D	Mean	St. D
Age (years)	80.7	8.7	85.1	7.0	81.4	8.6
Number of ambulance staff on scene (SWASFT)	2.7	1.4	2.7	1.7	2.7	1.4
Gender	n	%	n	%	n	%
Male	1,162	45.4	166	39.5	1,347	44.5
Female	1,396	54.6	254	60.5	1,680	55.5
Location**	n = 1,390	%	N = 246	%	N = 1,665	%
Home	1,126	81.0%	130	52.8%	1,275	76.6%
Care home	75	5.4%	103	41.9%	186	11.2%
Public place	118	8.5%	7	2.9%	126	7.6%
Other	71	5.1%	6	2.4%	78	4.7%

* includes the 49 patients excluded due to uncertainty as to whether dementia was recorded.

** 1,372 missing

For peer review only - http://bmjopen.bmj.com/site/about/guidelines.xhtml

Presenting condition and provisional diagnosis

The reason for the call (presenting condition) was less likely to be related to a cardiac or respiratory problem in the dementia group (4.8%; 95% CI: 3.0%, 7.3% and 5.8%; 95% CI: 3.7%, 8.4%) than the comparator group (10.8%; 95% CI: 9.6%, 12.1% and 11.2%; 95% CI: 10.0%, 12.5%), but more likely to be for a fall in the dementia group (15.6%; 95% CI: 12.2%, 19.4%) than in the comparator group (9.1%; 95% CI: 8.0%, 10.3%) (Figure 1). Similar findings were evident for the provisional diagnosis as recorded by the attending clinician. Patients with dementia were less often diagnosed with cardiac or respiratory problems (4.6%; 95% CI: 2.8%, 7.1% and 5.4%; CI: 3.4%, 8.0%) than patients in the comparator group (11.7%; 95% CI: 10.4%, 13.0% and 11.1%; 95% CI: 9.9%, 12.4%). Conversely, traumatic injuries and falls were more prevalent in the dementia group (20.0%; 95% CI: 16.2%, 24.1% and 11.4%; 95% CI 8.5%, 14.9%) than in the comparator group (13.5%; 95% CI: 12.2%, 14.9% and 7.3%; 95% CI: 6.3%, 8.4%).

Figure 1: Provisional Diagnosis (N = 2,848: 411 with dementia and 2,437 comparator group)

Social circumstances and frailty

Patients with dementia were more likely to be living in a care home and to have a care package in place (Figure 2). Only 6.7% (95% CI: 4.2%, 10.0%) of patients with dementia were recorded as living in their own home without a care package compared to 31.7% (95% CI: 29.5%, 33.9%) of the comparator group. Similarly, 12.4% (95% CI: 9.0%, 16.6%) of patients with dementia were living with extended family without a care package compared to 32.1% (95% CI: 30.0%, 34.3%) of the comparator group. 25.8% (95% CI: 21.0%, 31.0%) and 22.6% (95% CI: 18.1%, 27.6%) of patients with dementia were living in a nursing or residential home compared with 3.5% (95% CI: 2.7%, 4.5%) and 3.6% (95% CI: 2.8%, 4.6%) of the comparator group.

Figure 2: Living and social arrangements

Frailty scores were recorded by SWASFT in 1,128 patients of which 70.2% (95% CI: 63.1%, 76.6%) of patients in the dementia group were assessed as moderately to severely frail compared to 30.5% (95% CI: 27.6%, 33.6%) in the comparator group. Only 7.4% (95% CI: 4.1%, 12.2%) of patients in the dementia group were assessed as managing well or being very fit compared to 43.7 (95% CI: 40.5%, 47.0%) in the comparator group (Table 2).

For peer review only - http://bmjopen.bmj.com/site/about/guidelines.xhtml

Table 2: Frailty (N = 1128 with frailty data)

Frailty score	Comparator Group		Dementia Group	
	N	%	N	%
**Very Fit**	40	4.3	1	0.5
**Well**	137	14.6	2	1.1
**Managing well**	234	24.9	11	5.9
**Vulnerable**	111	11.8	18	9.6
**Mildly frail**	131	13.9	24	12.8
**Moderately frail**	147	15.6	43	22.9
**Severely Frail**	108	11.5	80	42.6
**Very severely frail**	15	1.6	6	3.2
**Terminally ill**	17	1.8	3	1.6
**Total**	940	100	188	100

Call cycle time and conveyance to hospital

The mean duration of the call (time of arrival on scene to time of closing the call) was shorter for people with dementia (85.2 minutes 95% CI: 81.5, 88.8) than for the comparator group (89.8 minutes 95% CI: 88.2, 91.5) (Table 3). In 61.0% of all cases the patient was conveyed to hospital by ambulance. Patients in the dementia group were less likely to be taken to hospital (50.4%); 95% CI: 45.5%, 55.2% compared to those in the comparator group (62.7; 95% CI: 60.8%, 64.6%). Fewer patients in the dementia group were recorded as experiencing pain (25.6%; 95% CI: 21.5%, 30.1%) than in the comparator group (39.5%; 95% CI: 37.6%, 41.4%).

Table 3: Call cycle time and conveyance to hospital (St. D = standard deviation; SWASFT = South Western Ambulance Service NHS Foundation Trust).

	Comparator		Dementia		Total	
	N = 2,567		N= 421		N=3,037	
	Mean	St D	Mean	St D	Mean	St D
Call cycle time in mins	89.8	41.3	85.2	38.3	89.4	40.9
	n	%	n	%	n	%
Number conveyed*	1,598/2,547	62.7	211/419	50.4	1,838/3,015	61.0

For peer review only - http://bmjopen.bmj.com/site/about/guidelines.xhtml

Number with Pain	1014	39.5	108	25.6	1132	37.3
Number with past medical history**	1,697/1,773	95.7	300/303	99.0	2,024/2,104	96.2

*22 missing

** SWAST only (207 missing)

Comorbidities

One or more comorbidities were recorded for 2,469 of the 3,037 (81.3%; 95% CI: 79.9%, 82.7%) cases. For patients in the dementia group, 352 of the 421 cases (83.6%; 95% CI: 79.7%, 87.0%) had at least one comorbidity recorded, and for the comparator group it was 2,076 of 2,567 cases (80.9%; 95% CI: 79.3%, 82.4%).

Respiratory and cardiac comorbidities were less likely to be recorded for patients with dementia, whereas neurological disorders, stroke and musculo-skeletal conditions were more prevalent (Figure 3). The presence of one or more comorbidities increased call cycle time and conveyance rate, irrespective of dementia (Table 4).

Figure 3: Comorbidities recorded

Table 4: Hospital conveyance and call cycle time with comorbidities

	**Conveyed to hospital**			**Call cycle time (minutes)**		
	**n**	**%**	**95% CI**	**n**	**mean**	**95% CI**
**Total**	1,838/3,015	61.0%	(59.2%, 62.7%)	2,996	89.4	(87.9, 90.9)
**Comparator**	1,598/2,547	62.7%	(60.8%, 64.6%)	2,532	89.8	(88.2, 91.5)
**Comparator (no comorbidities)**	273/481	56.8%	(52.2%, 61.2%)	480	82.8	(79.1, 86.6)
**Comparator (with comorbidities)**	1,325/2,066	64.1%	(62.0%, 66.2%)	2,052	91.5	(89.7, 93.3)
**Dementia**	211/419	50.4%	(45.5%, 55.2%)	418	85.2	(81.5, 88.8)
**Dementia (no comorbidities)**	33/69	47.8%	(35.6%, 60.2%)	69	78.7	(70.5, 86.9)
**Dementia (with comorbidities)**	178/350	50.9%	(45.5%, 56.2%)	349	86.4	(82.3, 90.5)

For peer review only - http://bmjopen.bmj.com/site/about/guidelines.xhtml

Discussion

Dementia was recorded in the Patient Care Record (PCR) in approximately 14% of cases. This finding is consistent with work carried out by Buswell et al,[16] which reported a rate of 14.5% in patients aged over 75 years.

Contrary to our hypotheses, patients aged over 65 years with dementia were less likely to be conveyed to hospital following an emergency call than patients over 65 years without dementia. In addition, the call cycle times were similar for patients regardless of whether or not they had dementia. This might indicate that the high A&E attendance rate for people with dementia observed in previous research [6,7] results from 'front door' attendances by people brought in by carers or relatives, rather than those conveyed to hospital by ambulance. Moreover, it is possible that because staff are aware of the negative outcomes associated with hospital admission for people with dementia, they avoid conveying these patients unless absolutely necessary.

One or more comorbidities were reported in the PCR in just over 80% of all cases, with little difference between the dementia group and those without dementia. This is incongruent with findings from other settings, where people with dementia have consistently been found to have a significantly higher incidence of comorbidity at hospitalisation [4,8] and in primary care.[5] This may be related to the fact that information recorded in ambulance records is obtained from sources on scene and is not linked reliably to other records of health care data. Reliable and accurate information about past medical history is not always available to ambulance staff. Nonetheless, when one or more comorbidities were recorded as present, patients were more likely to be conveyed to hospital. This difference was more evident in the comparator group than in the dementia group. In addition, call cycle times were longer for patients with comorbidities, regardless of the presence of dementia. It may therefore be deduced that call cycles last longer and conveyance rates are higher when older adults have complex needs or frailty associated with comorbidity; the presence of dementia is not necessarily an influencing factor in these outcomes.

Post-hoc analyses of conveyance to hospital and call cycle time were calculated on patients in different age bands (65-74, 75-84 and 85+ years). The findings indicate that in general, for younger people the call cycle time is longer and they are more likely to be conveyed to hospital. This is consistent with previous research on the epidemiology of non-conveyed patients, which found that older patients account for the majority of these calls. Moreover, falls account for 34%-40% of all non-conveyed patients.[17,18]

For peer review only - http://bmjopen.bmj.com/site/about/guidelines.xhtml

Dementia is associated with an increased risk of falls, and an increased level of fall related traumatic injury (31% vs 21%) was noted for this patient group. In previous research, Buswell et al [16] found falls to be the most common reason for an emergency call; in the present study, while a fall was the presenting complaint in over a quarter of cases overall, this proportion increased to almost half where dementia was recorded. Therefore, it is possible that the high number of falls in the dementia group accounts for the lower rate of conveyance. It was not possible in this research to establish which non-conveyance decisions were made by the attending clinician, and which were due to the patient's refusal to be transported, and this may also be a relevant factor. However, non-conveyance in older people who have fallen may be problematic since those not conveyed have been found to have a high rate of subsequent emergency healthcare contacts and an increased risk of death and hospital admission [18]. Although there is research ongoing to address prehospital assessment and management of people who have fallen [19], it is important to consider the impact of dementia in these protocols.

Of particular relevance to older people who have dementia, who have fallen and who are not conveyed is the assessment of pain; it is well recognised that pain is less often appreciated in dementia patients and is difficult to assess.[20,21] Despite the fact that we observed falls to be more common in the dementia group, pain was less often recorded in PCRs for those with dementia (25.6%) than for those in the comparator group (39.5%). More research is needed to establish reliable ways of assessing pain in people with dementia, to ensure that non-conveyance decisions are appropriate and do not lead to a further need for emergency healthcare.

Unsurprisingly, patients with dementia were more likely to be living in a care home (48%) than those without dementia (7%). Conveyance was more frequent for both groups when living in a care home than for those living in their own home; post-hoc analysis indicated that when in a care home, people with dementia are still less likely to be conveyed to hospital (54.6%) than those without (66.9%). Therefore, it is possible that care home staff do not always feel able to accurately assess these patients, and may rely on ambulance staff who, in turn, need to be equipped and skilled to reliably identify pain and assess other symptoms in people with dementia.

There are several limitations that impact on the generalisability of the findings and the conclusions that can be drawn from our study. Firstly, this was a retrospective study and there is the potential

For peer review only - http://bmjopen.bmj.com/site/about/guidelines.xhtml

for bias that may affect the selection of comparator cases. However, great care was taken to ensure data extraction was robust and this is detailed in the coding template. Secondly, the samples were only extracted from two services over two short periods. The time periods were in January and July to capture any seasonal variation. Additional issues that may have affected the reliability and validity of the data were associated with dementia diagnosis; recording of dementia on the PCR does not equate to a definitive diagnosis. Conversely, it is likely that some of the comparator cases had undiagnosed or unrecognised dementia. Nonetheless, in the absence of robust evidence in this field, this study provides both useful information and indications for further research.

Conclusion

The reason for a lower conveyance rate in patients recorded as having dementia remains unexplained. In this study comorbidity was a more accurate predictor of conveyance to hospital than dementia per se. We have, however, shown that ambulance services are called to people with dementia for different reasons than people without dementia. Therefore, it is possible that the lower conveyance rate for dementia patients may be associated with non-injury falls or transient symptoms that are not perceived as requiring admission to hospital. However, unless pain can be reliably assessed, decisions on non-conveyance for people with dementia may not necessarily be appropriate. In addition, care and nursing staff may have low thresholds for calling an ambulance for an individual that requires primary care management rather than conveyance to hospital. This would offer an explanation for the similar call cycle times we observed for both patients groups. However, further research is required to examine these issues in more detail.

This study has shown that higher rates of hospital admission for older people may be more related to complex needs and frailty than dementia. Given the unexpected nature of our findings, further research is required to understand how people with dementia interact with ambulance services, and the way in which ambulance clinicians make decisions at scene. There is much evidence to suggest that hospital admission for people with dementia results in adverse patient outcomes, and should be avoided where possible. However, evidence also suggests that patients who are not conveyed following a fall are at risk of poorer outcomes. Research on the assessment of pain and injury following a fall in people with dementia is a high priority, to ensure the most appropriate conveyance decisions are made.

For peer review only - http://bmjopen.bmj.com/site/about/guidelines.xhtml
For peer review only

Acknowledgments:

The authors would like to thank members of the Study Steering Committee for their contribution to this research: Professor Steve Iliffe (Professor of Primary Care for Older People, University College London); Professor Philp Bath (The Stroke Association Professor of Stroke Medicine, Chair and Head of the Division of Clinical Neuroscience and NIHR Senior Investigator, The University of Nottingham), Elinor Griffiths (Research Grants Manager, University Hospitals Bristol NHS Foundation Trust); Simon Goodwin (Programme Manager, Central Commissioning Facility, NIHR); Alan Bradley and John Long (Patient and Public advisors).

Funding statement:

This report is independent research funded by the National Institute for Health Research (Research for Patient Benefit Programme, Home or hospital for people with dementia and one or more other multimorbidities: what is the potential to reduce avoidable emergency admissions?, PB-PG-0215-36098). The views expressed in this publication are those of the author(s) and not necessarily those of the NHS, the National Institute for Health Research or the Department of Health.

Author contributions:

SV, JB, SB, MB, RC, SC, KK, SP and CS were involved in the conception and design of the study. SV, JB, JBr, SB, MB, RC, SC, TF, KK, LP, SP and CS made substantial contributions to development of the methodology and analysis. SV drafted the manuscript. JBr, TF, KK and LP led on the data collection and HT conducted the data analysis. All authors contributed to the critical revision of the manuscript for publication and approved the final version to be published.

Competing interests:

All authors have completed the ICMJE uniform disclosure form at www.icmje.org/coi_disclosure.pdf and declare: no support from any organisation for the submitted work; no financial relationships with any organisations that might have an interest in the submitted work in the previous three years; no other relationships or activities that could appear to have influenced the submitted work.

Data sharing statement:

For peer review only

For peer review only - http://bmjopen.bmj.com/site/about/guidelines.xhtml

Supplementary files and the dataset are available from sarah.voss@uwe.ac.uk.

For peer review only

For peer review only - http://bmjopen.bmj.com/site/about/guidelines.xhtml

REFERENCES

1. Alzheimer's Society (2014) https://www.alzheimers.org.uk/info/20027/news_and_media/541/facts_for_the_media (Accessed 17/01/2018)
2. NHS Digital (2016). Hospital Admitted Patient Care Activity, 2015-16. http://digital.nhs.uk/catalogue/PUB22378 (Accessed 17/01/2018)
3. Kings Fund (2017) Quarterly Monitoring Report (QMR) 22. http://qmr.kingsfund.org.uk/2017/22/ (Accessed 17/01/2018)
4. Zhao Y, Kuo T, Weir S, et al. Healthcare costs and utilization for Medicare beneficiaries with Alzheimer's. *BMC Health Serv Res* 2008;8(1):108.
5. Poblador-Plou B, Calderón-Larrañaga A, Marta-Moreno J, et al. Comorbidity of dementia: a cross-sectional study of primary care older patients. *BMC psychiatry* 2014;14(1):84.
6. Browne J, Edwards DA, Rhodes KM, Brimicombe DJ, Payne RA. Association of comorbidity and health service usage among patients with dementia in the UK: a population-based study. BMJ open. 2017 Mar 1;7(3):e012546.
7. Bunn F, Burn AM, Goodman C, Robinson L, Rait G, Norton S, Bennett H, Poole M, Schoeman J, Brayne C. Comorbidity and dementia: a mixed method study on improving healthcare for people with dementia (CoDem) *Health Serv Deliv Res* 2016*;4:1–156*
8. Phelan A, Borson S, Grothaus L, et al. Association of incident dementia with hospitalizations. *Jama* 2012;307(2):165-172.
9. Wittenberg R, Sharpin L, McCormick B, et al. Understanding Emergency Hospital Admissions of Older People. Centre for Health Service Economics and Organisation. Report 6, December 2014. See: https://www.chseo.org.uk/downloads/report6-emergencyadmissions.pdf
10. Buswell M, Lumbard P, Prothero L, et al. Unplanned, urgent and emergency care: what are the roles that EMS plays in providing for older people with dementia? An integrative review of policy, professional recommendations and evidence. *Emerg Med J*. 2014 Dec 2:emermed-2014.. Emergency Medicine Journal, emermed-2014.
11. Arnetz JE, Hamblin L, Essenmacher L, Upfal MJ, Ager J, Luborsky M. Understanding patient-to-worker violence in hospitals: a qualitative analysis of documented incident reports. *Journal of advanced nursing*. 2015 Feb 1;71(2):338-48.
12. Hodge A, Marshall A. Violence and aggression in the emergency department: A critical care perspective. *Australian Critical Care* 2007;20(2):61-67.
13. Gitlin L, Winter L, Burke J. Tailored activities to manage neuropsychiatric behaviors in persons with dementia and reduce caregiver burden: a randomized pilot study. *Am J Geriatr Psychiatry* 2008;16(3):229-239.
14. Sampson EL, Blanchard MR, Jones L, Tookman A, King M. Dementia in the acute hospital: prospective cohort study of prevalence and mortality. The British Journal of Psychiatry. 2009 Jul 1;195(1):61-6.
15. Alzheimer's Society. Counting the Cost: Caring for people with dementia on hospital wards. Alzheimer's Society; 2009.
16. Buswell M, Lumbard P, Fleming J, et al. Using ambulance service PCRs to understand 999 call-outs to older people with dementia. *Journal of Paramedic Practice* 2016;8(5):246-251.
17. Mikolaizak AS, Simpso PM, Tiedemann A, Lord SR, Close JC. Systematic review of non-transportation rates and outcomes for older people who have fallen after ambulance service call-out. *Australas J Ageing*. 2013; 32(3):147–157.

For peer review only - http://bmjopen.bmj.com/site/about/guidelines.xhtml

18. Snooks HA, Halter M, Close JC, Cheung WY, Moore F, Roberts SE. Emergency care of older people who fall: a missed opportunity. BMJ Quality & Safety. 2006 Dec 1;15(6):390-2.
19. Snooks H, Anthony R, Chatters R, Cheung WY, Dale J, Donohoe R, Gaze S, Halter M, Koniotou M, Logan P, Lyons R. Support and assessment for fall emergency referrals (SAFER 2) research protocol: cluster randomised trial of the clinical and cost effectiveness of new protocols for emergency ambulance paramedics to assess and refer to appropriate community-based care. BMJ open. 2012 Jan 1;2(6):e002169.
20. Lord B. Paramedic assessment of pain in the cognitively impaired adult patient. *BMC Emerg Med* 2009;9(1):20.
21. Care Quality Commission. Cracks in the Pathway. People's Experiences of Dementia Care as They Move between Care Homes and Hospitals. Newcastle upon Tyne. 2014;Care Quality Commission.

For peer review only

For peer review only - http://bmjopen.bmj.com/site/about/guidelines.xhtml

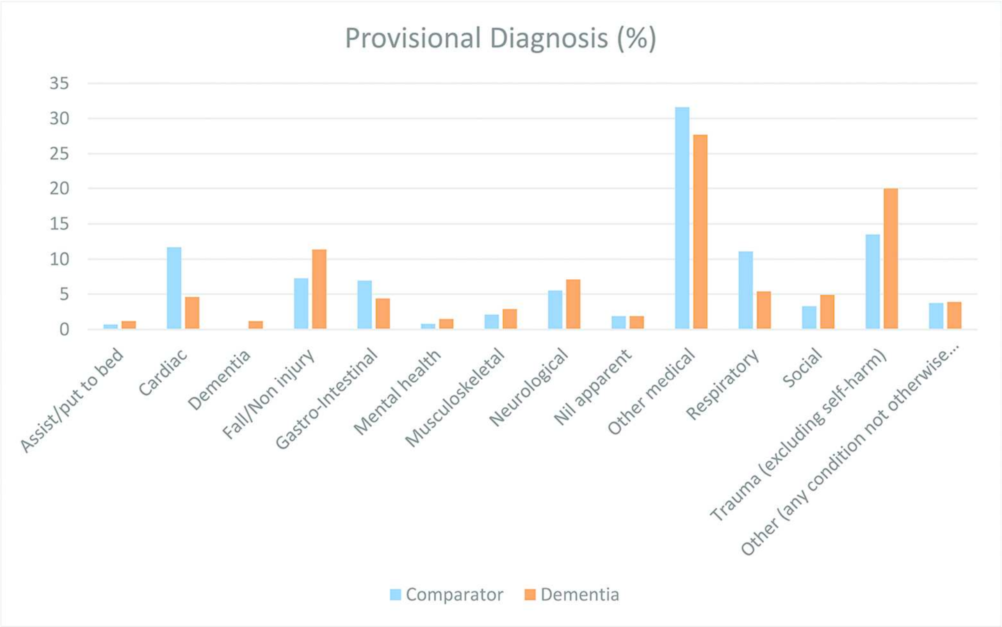

Figure 1: Provisional Diagnosis (N = 2848, N = 411 with dementia and N = 2437 for comparator)

128x90mm (300 x 300 DPI)

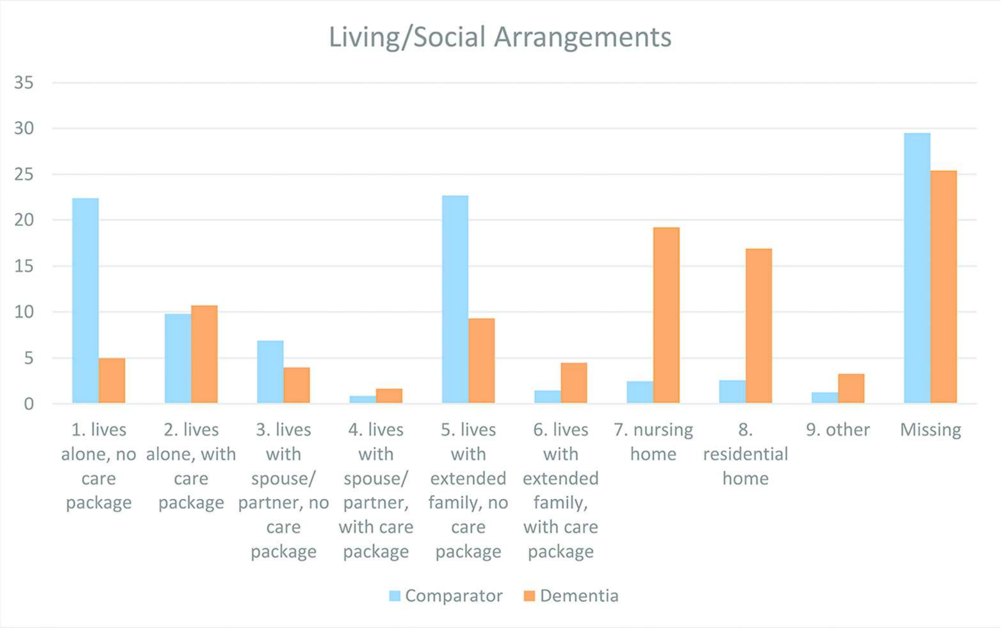

Figure 2: Living social arrangements
Note: data missing for 29.5% of comparator group and 25.4% of dementia group

128x90mm (300 x 300 DPI)

For peer review only

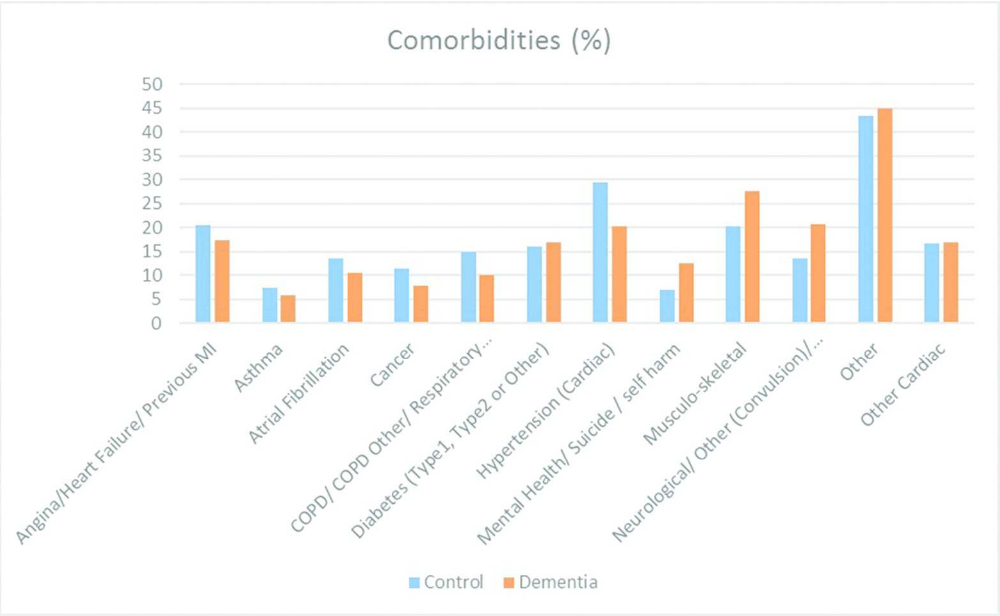

Figure 3: Comorbidities recorded

128x90mm (300 x 300 DPI)

Appendix A: HOMEWARD Phase 1- Coding for SWASFT/ EEAST Data

CODES

Call Colour codes:

1- red
2- green
3- amber- not available in EEAST

See Treat or Convey codes

1- See & Treat
2- See & Convey (include transport only in EEAST)

Qualification codes- identify most senior clinician recorded at scene

1. Technician
2. Paramedic/SWAMP paramedic
3. Lead Paramedic
4. Specialist Paramedic/Critical Care Paramedic
5. Other- contains the following
    a. BLS trained (first responder)
    b. Ambulance Care Assistant
    c. Emergency Care Assistant
    d. Student Paramedic
    e. Operational Officer
    f. Doctor

Gender codes

1- Male
2- Female

Presenting condition codes

SWASFT	EEAST
1- General	Other
2- Cardiac	AF, Heart Failure, Hypertension, MI, other cardiac
3- Respiratory	Asthma, COPD, other respiratory
4- Endocrine	Diabetes I or II, also other for thyroid
5- Neurological	Stroke/ CVA, TIA, Other neurological
6- Gastro-Intestinal	Other
7- Musculoskeletal	Musculoskeletal
8- Obs/gynae	Other
9- Cancer	Other
10- Mental health	Mental Health
11- Trauma (excluding self-harm)	Trauma
12- Other medical	Other
13- Social	Social
14- Other (any condition not otherwise specified)	Other

For peer review only - http://bmjopen.bmj.com/site/about/guidelines.xhtml

15- Deliberate self-harm	Suicide/ self-harm
16- Environmental	Other
(17- Sepsis) see other medical	Other e.g. UTI, Chest Infection
18- Poisoning (accidental)	Other
19- Burns	Other
	Cognitive impairment/ confusion- delirium
	Dementia
	Nil apparent
	Palliative

Provisional diagnosis codes

SWASFT	EEAST
1- General	Other
2- Cardiac	AF, Heart Failure, Hypertension, MI, other cardiac
3- Respiratory	Asthma, COPD, other respiratory
4- Endocrine	Diabetes I or II, also other for thyroid
5- Neurological	Stroke/ CVA, TIA, Other neurological
6- Gastro-Intestinal	Other
7- Musculoskeletal	Musculoskeletal
8- Obs/gynae	Other
9- Cancer	Other
10- Mental health	Mental Health
11- Trauma (excluding self-harm)	Trauma
12- Other medical	Other
13- Social	Social
14- Other (any condition not otherwise specified)	Other
15- Deliberate self-harm	Suicide/ self-harm
16- Environmental	Other
(17- Sepsis) see other medical	Other e.g. UTI, Chest Infection
18- Poisoning (accidental)	Other
19- Burns	Other
	Cognitive impairment/ confusion- delirium
	Dementia
	Nil apparent
	Palliative

For peer review only

For peer review only - http://bmjopen.bmj.com/site/about/guidelines.xhtml

Search terms for target and control groups:

Target group:

Dementia, Alzheimer's, Fronto-temporal dementia, vascular dementia, senility, Pick's disease, Lewy Body disease/ dementia

1- Dementia present - Presence of the diagnosis of one of the above
2- Dementia suspected - paramedic suggests dementia may be present eg ? dementia, seeing memory nurse, taking dementia medication
3- Dementia inferred - Paramedic infers ongoing problem through use of other language such mild cognitive impairment, cognitive impairment, memory loss. Sometimes this might also be called confusion but is <u>not</u> acute. This might be confirmed as 'normal or usual for the patient'.

Control group:

Confusion- acute, delirium,

4- Presence of one of the above

Drugs search terms:

Drugs: Acumor (XL), **Aricept**, Axurer, Consium XL, **Donepezil,** Ebixa, Elmino, Exelon, **Galantamine,** Galsya (XL), Gazylan XL, Gatalin (XL), Kerstipon, Lotprosin XL, Luventa XL, Maruxa, **Memantine,** Nemdatine, Nimvastid, Reminyl (XL), **Rivastigmine**, Valios.

PATCHES: Alzest, Eluden, Prometax, Rivatev, Voleze

1- Presence of these drugs in case record

Social History code:

1- lives alone, no care package
2- lives alone, with care package
3- lives with spouse/ partner, no care package
4- lives with spouse/ partner, with care package
5- lives with extended family, no care package
6- lives with extended family, with care package
7- nursing home
8- residential home
9- other

Location codes:

1- Home
2- Nursing home
3- Residential Home
4- Public place
5- Other
6- Not documented

For peer review only

For peer review only - http://bmjopen.bmj.com/site/about/guidelines.xhtml

Capacity to consent

1- Patient has capacity
2- Patient does not have capacity

Rockwood Frailty code:

1- Very Fit
2- Well
3- 3 Managing Well
4- Vulnerable
5- Mildly Frail
6- Moderately Frail
7- Severely Frail
8- Very Severely Frail
9- Terminally Ill

Falls

Search conducted in presenting condition and HPC for fall related calls. This is to ensure those coded later as trauma are also coded for falls. Falls are coded as present if they occurred within last 6 days and have contributed to the call.

Search terms fall, fell, slip, trip

For peer review only

For peer review only - http://bmjopen.bmj.com/site/about/guidelines.xhtml

STROBE Statement—Checklist of items that should be included in reports of ***cross-sectional studies***

	Item No	Recommendation	Page Number
**Title and abstract**	1	(*a*) Indicate the study's design with a commonly used term in the title or the abstract	1
		(*b*) Provide in the abstract an informative and balanced summary of what was done and what was found	2
**Introduction**			
Background/rationale	2	Explain the scientific background and rationale for the investigation being reported	5
Objectives	3	State specific objectives, including any prespecified hypotheses	5
**Methods**			
Study design	4	Present key elements of study design early in the paper	6
Setting	5	Describe the setting, locations, and relevant dates, including periods of recruitment, exposure, follow-up, and data collection	6
Participants	6	(*a*) Give the eligibility criteria, and the sources and methods of selection of participants	6
Variables	7	Clearly define all outcomes, exposures, predictors, potential confounders, and effect modifiers. Give diagnostic criteria, if applicable	6
Data sources/ measurement	8*	For each variable of interest, give sources of data and details of methods of assessment (measurement). Describe comparability of assessment methods if there is more than one group	*6*
Bias	9	Describe any efforts to address potential sources of bias	6
Study size	10	Explain how the study size was arrived at	6
Quantitative variables	11	Explain how quantitative variables were handled in the analyses. If applicable, describe which groupings were chosen and why	6
Statistical methods	12	(*a*) Describe all statistical methods, including those used to control for confounding	6
		(*b*) Describe any methods used to examine subgroups and interactions	6
		(*c*) Explain how missing data were addressed	6
		(*d*) If applicable, describe analytical methods taking account of sampling strategy	6
		(*e*) Describe any sensitivity analyses	6
**Results**			
Participants	13*	(a) Report numbers of individuals at each stage of study—eg numbers potentially eligible, examined for eligibility, confirmed eligible, included in the study, completing follow-up, and analysed	8-11
		(b) Give reasons for non-participation at each stage	8-11
		(c) Consider use of a flow diagram	N/A
Descriptive data	14*	(a) Give characteristics of study participants (eg demographic, clinical, social) and information on exposures and potential confounders	8-11
		(b) Indicate number of participants with missing data for each variable of interest	8-11
Outcome data	15*	Report numbers of outcome events or summary measures	8-11
Main results	16	(*a*) Give unadjusted estimates and, if applicable, confounder-adjusted estimates and their precision (eg, 95% confidence interval). Make clear which confounders were adjusted for and why they were included	8-11

For peer review only

		(*b*) Report category boundaries when continuous variables were categorized	8-11
		(*c*) If relevant, consider translating estimates of relative risk into absolute risk for a meaningful time period	8-11
Other analyses	17	Report other analyses done—eg analyses of subgroups and interactions, and sensitivity analyses	8-11
**Discussion**			
Key results	18	Summarise key results with reference to study objectives	12
Limitations	19	Discuss limitations of the study, taking into account sources of potential bias or imprecision. Discuss both direction and magnitude of any potential bias	13-14
Interpretation	20	Give a cautious overall interpretation of results considering objectives, limitations, multiplicity of analyses, results from similar studies, and other relevant evidence	12-14
Generalisability	21	Discuss the generalisability (external validity) of the study results	14
**Other information**			
Funding	22	Give the source of funding and the role of the funders for the present study and, if applicable, for the original study on which the present article is based	15

*Give information separately for exposed and unexposed groups.

Note: An Explanation and Elaboration article discusses each checklist item and gives methodological background and published examples of transparent reporting. The STROBE checklist is best used in conjunction with this article (freely available on the Web sites of PLoS Medicine at http://www.plosmedicine.org/, Annals of Internal Medicine at http://www.annals.org/, and Epidemiology at http://www.epidem.com/). Information on the STROBE Initiative is available at www.strobe-statement.org.

For peer review only
